# Life style and Parkinson’s disease

**DOI:** 10.1007/s00702-022-02509-1

**Published:** 2022-05-23

**Authors:** Heinz Reichmann, Ilona Csoti, Jiri Koschel, Stefan Lorenzl, Christoph Schrader, Juergen Winkler, Ullrich Wüllner

**Affiliations:** 1 Department of Neurology, University Hospital Dresden, Fetscherstrasse 74, 01307 Dresden, Germany; 2 Fachklinik für Parkinson, Gertrudis Klinik Biskirchen, Karl-Ferdinand-Broll-Straße 2-4, 35638 Leun-Biskirchen, Germany; 3 Parkinson-Klinik, Ortenau GmbH & Co. KG, Kreuzbergstraße 12-16, 77709 Wolfach, Germany; 4 Neurologie und Palliative Care, Krankenhaus Agatharied, Norbert-Kerkel-Platz, 83734 Hausham, Germany; 5 Neurologische Klinik mit Klinischer Neurophysiologie OE 7210, Carl-Neuberg-Str. 1, 30625 Hannover, Germany; 6 Zentrum für Bewegungserkrankungen, Molekulare Neurologie, Universitätsklinikum Erlangen, Schwabachanlage 6, 91054 Erlangen, Germany; 7 Department of Neurology, University Clinic Bonn and German Center for Neurodegenerative Diseases (DZNE), 53127 Bonn, Germany

**Keywords:** Parkinson’s disease, Environment, Physical activity, Nicotine, Caffeine, Alcohol, Life style

## Abstract

The question whether life style may impair the advent or course of the disease in patients with Parkinsonism is of great importance for patients and physicians alike. We present here comprehensive information on the influence of the environment, diet (especially caffeine, nicotine, alcohol, chocolate and dairy products), physical activity and sleep on risk and course of Parkinson’s disease.

## Introduction

The question as to whether or not the course of Parkinson’s disease (PD) can be modified by life style adjustments remains largely unanswered. On the other hand, increasing numbers of people strive to adjust their life styles to prevent dementia, obesity, diabetes or hypertension. Amongst the elderly, there is particular concern about diseases such as PD or Alzheimer’s disease.

Therefore, we here discuss life style measures and their possible impact on risk reduction or prevention of PD and putative improvement of motor or non-motor symptoms in PD patients with a special emphasis on pesticide exposure and environment, physical activity, sleep and dietary aspects, but did not go into detail with respect to an analysis of the increasing data regarding the microbiome in PD and putative effects of pre- and probiotics. Many measure intermingle and the various effects which could modulate the immune system and may, thus, exert indirect effect upon PD symptoms have not yet been investigated.

## Pesticide exposure and environment

**Fig. 1:**
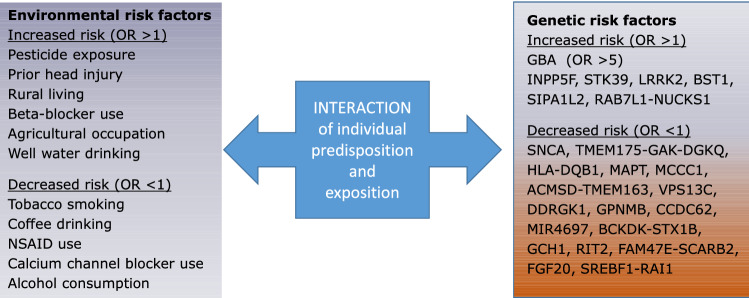
Interplay between predisposition and exposition for the development of Parkinson's disease

The current view of the pathogenesis of PD comprises the interaction of a genetic background with environmental factors (dual factor theory) (Fig. 1). Environmental toxins such as air pollution, metal ion contamination and pesticide exposure are regarded potential risk factors as they cause oxidative stress, mitochondrial dysfunction and chronic neuro-inflammation (Nandipati and Litvan 2016). These factors have been discussed in numerous research papers and accumulating evidence suggests that they are strongly associated with the risk to develop PD. For decades, pesticides and fertilizers have been utilized to improve the fertility of farmland a high incidence of PD has been observed not only in chemical workers involved in the preparation of these pesticides, but also in farmers and the general population living in rural areas and consuming well water. The effect of pesticide exposure has been demonstrated in studies and meta-analysis around the world. Recently, the use of paraquat and rotenone was shown to be associated with an increased risk of developing PD (Brouwer et al. 2017; Liu et al. 2020) and prolonged pesticide exposure was associated with an 11% increase in PD (Yan et al. 2018). In France, PD is considered an occupational disease of farmers (Ahmed et al. 2017; Delamarre and Meissner 2017). In addition to paraquat and rotenone, organophosphates, organochlorides, pyrethroids and triazines have been associated with an increased risk of PD. Thus, a wide range of pesticides used in the past and still in use today have the potential to increase the risk of developing PD. Given the sometimes-long half-life of these chemicals and the possibility of accumulation in food chains, the risk might be even higher than the studies suggest. Given the potential harmful effects to dopaminergic neurons, animal models of PD have been set up using pesticides such as rotenone to induce substantia nigra neuronal death. Similar to farmers who inhale the pesticides, rotenone was effective when administered via the nasal cavity. In several studies, an association between pesticide exposure and variants in PD-associated genes have been identified, suggesting that pesticide exposure leads to dysregulation of specific miRNAs, which might also explain their long-term effect (Liu et al. 2020).

Another positive correlation with the prevalence of PD has also been demonstrated for industrialization (Mezynska and Brzoska 2018; Anyanwu et al. 2018). In particular, the occupational exposure to metal ions like Pb, Cu, Fe, Al, Hg and Mn may increase the risk of PD (Bellou et al. 2016). These metal ions also occur in water and food and might be part of air pollution. While metal ions are essential for enzyme activity and are thus critically important, overdosage or overexposure is harmful. Altered enzyme function can induce mitochondrial dysfunction and oxidative stress and metal ion exposure impacts the aggregation properties of α-synuclein (α-syn) (Bisaglia et al. 2009).

Conflicting results have been published for air pollution, which in itself needs to be defined with regard to the presumed pollutants. The effect of air pollution is particular difficult to analyze, as a variety of factors influence the pollution that is also dependent on local climate changes. In a recent meta-analysis of “regulated” air pollution, i.e., PM2.5, NO2, O3 and CO concentration did not seem to exert a significant effect (Kasdagli et al. 2019).

## Physical activity

Prevention of neurodegenerative diseases will be one of the most important challenges in the future, given the very rapid growth of aging populations in eastern and western industrialized countries. Present professional occupational activity is characterized by reduced physical activity, which in conjunction with other environmental factors, may have been responsible for the increased prevalence of PD in the last 2 decades. Clinically, it is well accepted that activating therapies such as physiotherapy, ergotherapy and speech therapy, in addition to medication, constitute important corner stones of a comprehensive therapeutic approach. There are numerous studies showing that **“**physical activation” improves muscle strength, balance, gait, posture, cognition, general well-being and quality of life measures (Gaßner et al. 2019). In addition, motor scores such as the UPDRS-III improve with increased physical activity and recent data showed an impact also on mortality rate (Yoon et al. 2021. Furthermore, special training based on Lee Silverman Voice Treatment (LSVT), is able to improve the UPDRS motor score and mobility at home, as well as quality of life (Ebersbach et al. 2015).

However, the most important question of whether increased physical activity may delay the onset, or, even, prevent the onset of PD is still a matter of debate. The initial study by Sasco et al. (1992) and the long-term follow-up study by Logroscino et al. (2006) focused on this challenging question in their investigations in Harvard alumni. Based on questionnaires and death certificates, a partial positive protective tendency was observed. A protective effect against developing PD was observed in male participants who walked a distance of 5–10 km/week. At present, there are very large cohort studies, such as the *Health Professionals Follow Up Study* (HPFS) (Chen et al. 2005), the Cancer Prevention Study II nutrition (Thacker et al. 2008) and the *Diet and Health Study from the National Institutes of Health and American Association of Retired Persons* (NIH-AARP) (Xu et al. 2010), which strongly indicate a positive effect of physical activity for lowering the risk of PD. All three prospective studies are based on questionnaires, and show a significant protective effect, solely for male participants with a history of intensive physical activity of more than ten months between the ages of 30–50 years. These large cohorts were reanalyzed in the meta-analysis of Fang et al. (2018), covering a total of 8 prospective studies with more than half a million participants including some 2200 PD patients and a median follow-up period of 12 years. This meta-analysis showed a reduced risk to develop PD associated with the highest level of either total physical activity or moderate to vigorous physical activity. It is important to note though, that light physical activity does not suffice to lower the risk. To better quantify physical activity, the authors attempted to assess the risk based on metabolic equivalent of task per hour (MET/h). For instance, one-hour walking encompassing 5 km/h reflects 4 METs/h, one-hour cycling at a speed of 25 km/h 10 METs/h, respectively. Based on this meta-analysis, the authors provided a dose-dependent effect of physical activity for the future risk of PD showing that 30 METs/h per week of moderate–high intensity reduces the risk by about 50%. In real-life scenarios, 3 h of cycling at a speed of 25 km/h, thus, supposedly decreases the risk of PD by about 50%. In terms of walking, it would indicate that a person must walk at least 1 h/day at a speed of 5 km/h to decrease the risk to develop PD by 50%. It is evident that during the premotor phase of sporadic PD, physical activity declines; however, most of the cited prospective studies tried to control for this effect by excluding patients in the early disease phase. It is important to note that there is no dose-dependent response association between physical activity and PD risk in women; the association was observed in men only. This sex-specific effect is somewhat reminiscent to the effects of certain nutrients (dark berries, which exert positive effects in men only. These, as the effects of physical activity (while promising) may be too weak to exceed or outweigh the protective effects of estrogens.

## Sleep

It is well known that PD patients suffer from various sleep disorders, i.e., disease-related sleep disorders due to nocturnal dopamine deficiency or side effects of PD medication, but also typical concomitant diseases, such as restless legs syndrome or sleep apnea, must be taken into account in diagnosis and therapy. REM sleep-related behavior disorder (RBD) often precedes the motoric and, thus, symptomatic phase of the disease by years (Taguchi et al. 2020). It is tempting to speculate that specific sleep patterns may be associated with neurodegeneration. On the other hand, whether an improvement in sleep hygiene could prevent or slow down the progression of the disease.

In their prospective study, Chen et al. (2006) surveyed 84,794 nurses regularly over several years (1988–2000) on various aspects of their health (US Nurses’ Health Study). At the end of the study period, 181 cases of Parkinson's were recorded among the respondents. Contrary to expectations, the incidence of PD decreased steadily with the number of years of working night shifts and average sleep duration was positively correlated with the risk of PD. However, these findings should be interpreted with extreme caution. It cannot be concluded from correlations that shift work "protects" against PD, nor that long sleeping hours foster PF. Other factors with known correlation to PD risk such as use of estrogen replacement, aspirin or other non-steroidal anti-inflammatory drugs, tobacco and caffeine consumption were not identified. Indeed, the authors pointed to these limitations themselves and noted that shift work and disturbed sleep are associated with various diseases (such as gastrointestinal disorders, cardiovascular diseases, diabetes and certain types of cancer). Indeed, these findings were not confirmed in a similar study with Danish nurses, where no associations were found between incidences of PD and different shift work hours, including night shift, or persistency of shift work (Therming Jørgensen et al. 2020).

In addition to RBD, chronic insomnia may increase the risk of PD. Hsiao et al. (2017) compared 91,273 patients with sleep disorders (excluding apnea) to a similar number of controls with regard to the risk of developing PD and found that sleep disorders were independent risk factors for developing PD: patients with chronic insomnia lasting longer than three months had the highest risk (Hsiao et al. 2017). This was confirmed by Lysen et al. (2019), who argued that deterioration of sleep quality and duration are markers of the prodromal phase of Parkinsonism, including PD. Already suffering from PD, a link between insomnia and neurodegeneration is likely. The presumed mechanisms include nocturnal oxygen deprivation, inflammatory processes, impaired glymphatic clearance, abnormal proteostasis, and altered modulation of specific neuronal circuits during sleep that promote preexisting alpha-synucleinopathy (Lysen et al. 2019). However, more research is required to elucidate the relationship between sleep disorders and the development of PD in detail to find out whether, how and under which circumstances (e.g., genetic or other dispositions) sleep disorders may even causally trigger neurodegenerative processes.

## Diet

Epidemiological studies suggesting a positive association of particular dietary items and metabolic profiles with PD risk, especially in men, have been reported for caffeine, increased uric acid and total cholesterol levels (Ascherio and Schwarzschild 2016). While mediterranean diet has been associated with the risk of PD, limited research has been performed on other particular types of diet (Strikwerda 2021). Strikwerda and colleagues studied the relationship between overall diet quality and PD risk in the general Dutch population prospectively in 9414 participants from the Rotterdam Study. After an average follow-up of 14 years, PD was diagnosed in 129 participants and the results corroborated previous findings of a possible protective effect of a Mediterranean diet. Similarly, in a Swedish cohort study, higher adherence to a Mediterranean diet in middle age was associated with lower risk of PD (Yin 2021). Thus, increasing evidence supports the recommendation of a diet with "balanced" seasonal, regionally sourced fresh products, with a focus on fruit and vegetables and low-processed food with only a small proportion of simple carbohydrates for the prevention of PD (Csoti 2018). On the other hand, experimental studies suggest that nutrition and metabolic conditions such as obesity and type 2 diabetes may increase the risk of PD after middle age (Nam 2018). At present, the effects of a specific diet on the intestinal microbiota and human metabolism are ill understood, as microbiota only recently emerged as a detrimental factor for human health in general and PD risk and course in particular (Bedarf 2017). Recent evidence suggests that gut microbes may be involved in the seeding of prion-like proteins and their subsequent dissemination to the central nervous system and also shows strong interactions with neurons and glia, deeply affecting their function and behavior in health and disease. These implications are not the scope of this article and have been reviewed in Gentile et al. (2020) and Boulos and co-workers (2019).

## Chocolate consumption

Clinical observations amongst in-patients with PD in Dresden suggested an increased chocolate consumption. We speculated that due to its high content of biogenic amines chocolate may partially substitute the dopaminergic system and may improve PD symptoms. In a structured interview, 498 PD patients and their partners were questioned about their consumption of chocolate and other sweets. Questionnaires from 274 PD patients (55%) and 234 controls were eligible for further analyses (Wolz et al. 2009). The main outcome of this study was the observation of significant higher chocolate consumption in PD patients compared to their partners. It was speculated that ingredients such as phenylethylamine and caffeine analogues might be responsible for this preference. In a second study, normal chocolate was compared with cacao-free white chocolate with respect to improvement in motor symptoms in PD patients (Wolz et al. 2012). One hour after intake of dark chocolate, the UPDRS-III improved significantly, which was not the case for white chocolate. After 3 h, there was no significant difference. ß-phenylethylamine blood levels were unaltered in blood. As this study was a pilot in only 26 participants, further analyses are needed.

## Nuts

Nuts contain high concentration of monounsaturated fatty acids (MUFA) and polyunsaturated fatty acids (PUFA) and a low concentration of saturated fats. Furthermore, some nuts, particularly walnuts, are rich food sources of α-linolenic acid, a plant-based n-3 fatty acid. Additionally, nuts are substantial food sources of fiber, B-vitamins, minerals, and antioxidant compounds like alkyl phenols. Peanuts, although botanically classified as legumes, present with a similar nutrient profile as tree nuts and are therefore commonly included in this group (Bolling 2011). Eating nuts has significant beneficial impact on a variety of cardiovascular risk factors such as blood glucose, LDL, HDL, Apo B/Apo A-1 ratios, and even though they have a high caloric density, weight gain is rarely observed, in fact, nuts may even promote weight loss (de Souza 2017). Nuts thereby may improve hypertension, cardiovascular disease, diabetes type 2, reduce the risk of cancer and cognitive decline, the latter probably by optimizing the cerebrovascular risk profile on the one hand, and maybe by anti-oxidative neuroprotective properties on the other (Ros 2021).

In a rotenone rat model of PD, feeding cashew nuts, which are rich in alkyl phenols, resulted in less rotenone-induced behavioral changes and less oxidative stress. These effects were in part ascribed to a modulatory action on the mitochondria and SOD gene expression. These data suggest that alkyl phenols from nut might have neuroprotective action against degenerative changes in PD (Medeiros-Linard 2018). Prospective studies exploring the impact of nuts on the risk of developing PD are lacking, and their effect on disease progression of manifest PD has not yet been examined. It is fair, however, to assume that their ability to mitigate the impact of comorbidities and possible anti-oxidative properties may be beneficial.

## Dairy products

The 2015 Dietary Guidelines for Americans (DGA) identified dairy products as key contributors for calcium, phosphorus, vitamin A, vitamin D, riboflavin, vitamin B12, protein, potassium, zinc, choline, magnesium, and selenium intake (HHS Scientific Report, 2015). Subsequently, the DGA recommended 3 servings of dairy products per day for adults (one serving being 250 ml milk, 200 ml yoghurt, 40 g cheese, or 120 g ricotta cheese, respectively).

Consumption of dairy products and especially milk has been linked to an increased risk to develop PD (Chen 2002). Analyzing the dietary information from the HPFS and the NHS over a period of 12–18 years with respect to developing PD the authors found an 80% increased risk for PD in men that consumed > 2.9 servings of dairy products per day compared to those consuming < 1 serving per day. This increased risk was sex specific and not found in women.

Four large prospective cohort studies confirmed the association of milk and other dairy products, yet with a certain variability in hazard ratios. The Cancer Prevention Study found a higher risk among milk consumers in both men (80%) and women (50%) with an intake of > 502 g/day (~ 2 servings of milk) compared to those who drank 133.6 < g/day (Chen 2007). Other dairy products showed lower risks. The Honolulu Heart Program found, after further adjustment for dietary and other factors, a HR of 2.3-fold for developing PD (95% CI 1.3–4.1) in the highest milk intake group (> 16 oz/day; > 473 ml/day) vs those who consumed no milk (Park 2005). The Finnish Mobile Clinic Survey is the only study which found increased PD risk also for female milk consumers: women consuming > 613 g/day had a 3.31-fold increased risk of PD compared with women consuming < 370 g/day (Saaksjarvi 2013). The risk of men consuming milk > 950 vs 445 g/day was also increased, although not significantly. Taken together, the risk was 2.16-fold greater for those with the highest milk consumption opposed to those with the lowest. The Greek EPIC study examined a variety of dietary and lifestyle factors with respect to the risk of PD, but did not separate between men’s and woman’s risk. The most significant risk after adjustment for other factors was found for milk (34%), while no association was observed for cheese or yoghurt (Kyrozis 2013).

A meta-analysis of these five prospective cohorts stated a linear dose–response relationship showing that the risk for PD was increased by 17% for every 200 g milk/day and 13% for every 10 g cheese/day; consuming yoghurt and butter did not show any correlation (Jiang 2014). A re-examination of the PD risk in the HPFS and NHS cohorts differentiating between low- and high-fat dairy products showed significant differences. Consuming low-fat dairy (skim and low-fat milk, sherbet/frozen yoghurt, yoghurt, cottage cheese, and low-fat cheese) bore a pooled increased risk of 34% comparing < 1 vs ≥ 3 servings/day (Hughes 2017). For high-fat dairy foods (whole-fat milk, cream, ice cream, sour cream, butter, cream cheese, and other cheese), the associations tended to be in the opposite direction, i.e., a significant linear trend for decreased risk associated with greater intake of high-fat dairy. This study identified skim and low-fat milk as the main risk drivers. The results of this study were confirmed in a Swedish cohort which showed an increased risk for milk consumption of 29% for 201–400 ml/d vs less than 40 ml/d, but not for fermented milk products such as yoghurt or soured milk (Olsson 2020). A dose dependency for milk consumption as in the formerly published meta-analysis was not found.

In conclusion, consumption of milk consistently increased the risk of developing PD, and low-fat milk/skim milk/low-fat dairy products (and maybe cheese) seem to be the main drivers, while processed/fermented dairy products are possibly less problematic. Men seem to be more prone to the risk than women.

The mechanisms by which milk influences the risk of PD remains speculative. It is unlikely due to calcium, protein, vitamin D, lactose, or fat (Hughes 2017). One theory is that milk intake lowers uric acid and is thereby harmful (Dalbeth 2012; Vieru 2017). Other possible mechanisms include altered microbiome induced by dairy products or lactose intolerance which may contribute to intestinal inflammation and intestinal permeability, but this has not yet been thoroughly explored (Mischley 2019). A putative factor discussed initially is contamination with neurotoxic pesticides as, e.g., heptachlor epoxide that contaminated Hawaiian milk in the early eighties; however, this could not explain why the risk was highest in low-fat dairy products (Hughes 2017).

## Tea and coffee consumption

Coffee consumption is among the best studied nutritional habits in PD, while data on tea drinking are rather scarce; in particular, no data from randomized clinical trials have been released for green tea and a meta-analysis of tea drinking and risk of PD dates back to 2012 (Li 2012; Ascherio and Schwarzschild 2016; Chen and Schwarzschild 2020). The polyphenols theoflavin and epigallocatechin gallate, found in tea, are believed to have anti-oxidant, anti-apoptotic, and anti-inflammatory properties (Gosslau 2011). Based on the findings of numerous preclinical studies, green tea polyphenols have been evaluated for the treatment of de novo PD patients (Efficacy and Safety of Green Tea Polyphenol in De Novo Parkinson's Disease Patients, NCT00461942). The study has been completed, but as of yet, the results have not been reported. In the above-mentioned meta-analysis, eight articles including 1418 cases and 4250 controls were included. The pooled odds ratio (95% CI) was 0.85 (0.74–0.98) suggested a protective effect of tea drinking on PD risk, but there was no overt dose–response relationship (Li 2012).

In addition to caffeine, coffee contains, amongst other compounds, theophylines, various flavonoids, and tannins with antioxidant effects. In an open study of Parkinson's disease patients, non-motor symptoms improved with no sex difference (Altmann 2011). Several epidemiological studies found a greater effect with respect to PD risk in men compared to women, which suggests again, a week effect compared to the protective effect of being female (Costa 2010). Caffeine and urate are both purines, and have been investigated in the Harvard Biomarkers Study (566 subjects of idiopathic PD patients and healthy controls) (Bakshi 2020). Caffeine intake, as assessed by a validated questionnaire, was significantly lower in idiopathic PD patients compared to healthy controls in males (mean difference -125 mg/day, *p* < 0.001) but not in females (mean difference − 30 mg/day, *p* = 0.29). A strong inverse association was also observed with plasma urate levels both in males (mean difference − 0.46 mg/dL, *p* = 0.017) and females (mean difference − 0.45 mg/dL, *p* = 0.001). Both analyses stratified for sex and adjusted for age, body mass index, and either urate level or caffeine consumption, respectively. These results highlight the robustness of caffeine intake and urate as factors inversely associated with idiopathic PD.

Caffeine and synthetic adenosine A2A antagonists have been studied in phase II and III clinical trials for symptomatic treatment of PD (Barkhoudarian 2011). Istradefylline is an A2A adenosine receptor antagonist and an analog of caffeine; in 2013, it was approved in Japan at a dosage of 20 mg for therapy in PD under the trade name Nouryant/Nouriast®. After re-examining its initial opinion, the European Medicines Agency has confirmed its recommendation to refuse marketing authorization for the medicine Nouryant in November 2021, as only four out of the eight studies performed so far showed a reduction in ‘off’ time, and the effect did not increase with an increased dose of Nouryant. The Agency also noted that no effect was seen in the two studies which included patients from EU populations, including the most recent study, which involved patients who were receiving the maximum and optimal treatment for PD.

Reduced PD risk is strongly associated with caffeine consumption among men, the relationship is more complex in women, among whom caffeine’s overall link to PD is generally smaller or absent (Chen and Schwarzschild 2020). With regard to symptom control, however, the effects are similar, although the correlation of caffeine consumption by those already diagnosed with PD and their subsequent disease progression is less straightforward. Intriguingly, caffeine use has consistently been associated with reduced rates of developing levodopa-induced dyskinesias (LID): in the CALMPD trial cohort, early PD participants who reported higher daily intake of caffeine were less likely to develop dyskinesia after nearly six years of treatment (Wills 2013) and similar results were reported in an independent study in Italy (Nicoletti 2015).

## Alcohol

Studies in rodents connected alcohol intake and the induction of CYP2E1 to dopamine neurotoxic effects; the flavonoids resveratrol and quercetin contained in red wine exert anti-apoptotic and neuroprotective effects in animal models (Hu et al. 2006). Protective effects of resveratrol and quercetin against MPP+ -induced oxidative stress are mediated by modulation of apoptotic death of dopaminergic neurons (Bournival 2009). The quantity of red wine that would be required to test the postulated effects of resveratrol in humans, however, prohibits a clinical trial. There are no robust data for the commercially available resveratrol preparations, and liberal ingestion must, thus, be discouraged. Part of the neuroprotective effect of red wine has also been attributed to oligomeric proanthocyanidins, bioflavonoids found in grape seeds, (including catechin, epicatechin, epicatechin gallate, epigallogatechin-3-gallate) (Csoti 2018).

Unfortunately, the available data in humans are inconsistent. Some epidemiologic studies such as the Leisure World Cohort study (Paganini-Hill 2001) and the General Practice Research Data Base of the United Kingdom (Hernan et al. 2004) did not find an association between PD and alcoholic beverages. Paganini Hill studied a cohort of 13,979 residents of Leisure World Laguna Hills, a retirement community in California. Using questionnaires, death certificates and discharge diagnosis from hospitals, they identified 395 PD cases between 1981 and 1992. For each PD case, they age- and sex-matched 6 controls. The risk for PD was reduced by 20% in smokers, coffee drinkers and alcohol consumers. While there was a risk reduction in those who drank wine, beer and hard liquor if they had more than 2 glasses per day, this did not reach significance. In an epidemiological study of PD patients, no association was found between red wine consumption and MP (Palacios 2012).

The study of Hernan et al. (2004) is based on over 3 million Britons who were monitored by selected general practitioners during the 1995–2000 period. They defined alcoholics as those with such a diagnosis, or related chronic disease such as alcoholic cirrhosis, alcoholic cardiomyopathy or patients with more than 500 ml of ethanol per week. They selected 10 well-matched controls per case. The overall finding was that alcoholics had no higher or lower risk of developing PD than non-alcoholics. It is noteworthy however, that men had a slightly (nonsignificant) increased risk of developing PD. In addition, the authors did not find a dose–response relation between alcohol intake and PD. Thus, patients with an addiction (to alcohol) are not protected from PD.

In contrast, there are studies that claim that beer drinking may be beneficial (Hernan et al. 2003; Liu et al. 2013). Hernan et al. (2003) studied the association between drinking alcoholic beverages and PD in two large prospective cohorts, i.e., the Nurses’ Health Study and the Health Professionals’ Follow-up Study. Every two years, 121,700 female nurses and 51,529 male health professionals receive questionnaires including questions with respect to alcohol consumption. At their last follow-up, the authors detected 415 new PD cases. There was no association between wine and liquor consumption with PD; however, beer drinkers had a somewhat lower incidence of PD than non-drinkers did. The authors speculate that the 30% lower incidence after beer intake may be because beer increases serum uric acid levels. Uric acid is considered to offer protection by scavenging oxygen radicals. It is further noteworthy that this positive effect of beer drinking was found even with moderate beer consumption. Liu et al. (2013) report on 306,895 participants of the NIH-AARP Diet and Health Study in which they studied alcohol consumption in the years 2000–2006 and detected 1113 PD cases. Total alcohol consumption was not associated with a higher risk of developing PD.

In contrast to non-beer drinkers, beer drinkers had an odds ratio of 0.79—increased liquor consumption led to more PD. Increased wine consumption had a borderline lower risk for PD. From a mechanistic standpoint, beer might be positive as it increases urate levels, which was reported to be protective against the development of PD (Weisskopf et al. 2007).

An interesting study, reported by Eriksson et al. (2013), which consisted of follow-up of 36 years in patients who were admitted to hospitals in Sweden either because of alcohol-related problems or because of appendicitis, was conducted between 1972 and 2008 and during this period of time, 1741 PD patients were found, out of a cohort of 602,930 individuals. There was a 38% increased risk to develop PD in those with alcohol abuse disorders. The risk was especially high in young alcohol addicts. In a Finnish study, even those with a moderate alcohol consumption of less than 5 g per day had a higher risk to develop PD than non-drinkers did (Sääksjärvi et al. 2014). For alcohol in general, consumption of more than 5 g per week (i.e., approximately 0.5 L of wine) is most likely associated with an increased risk of dementia (Topiwala et al. 2017). Thus, the sevenfold increase reported in this study may reflect both Finnish drinking habits and the bias of severe alcohol dependency.

In a recent study by Peters et al. (2020), 220,494 participants from the so-called NeuroEPIC4PD study were analyzed and 694 incident PD cases were detected. The authors could not detect any association between baseline and lifetime total alcohol consumption and PD risk. Furthermore, they did not describe any special risk for beer, wine or liquor. There was also no difference between findings in German, Greek, Italian, Dutch, Spanish, Swedish and British patients.

Alcohol intake and PD risk was investigated in the million women study; during an average of 17.9 years of follow-up, 11,009 women had a new record of PD among 1309,267 women. In drinkers, the multivariable-adjusted relative risk comparing women who drank more than 14 drinks of alcohol per week with women who drank 1–2 drinks of alcohol per week was 0.99 (95% confidence interval 0.90, 1.10). These results do not support an association between alcohol intake and PD risk in women.

On the other hand, a recent Mendelian randomization study suggested causal associations of alcohol intake (OR 0.79; 95% CI 0.65–0.96; *p* = 0.021) and smoking continuation (which compares current vs. former smokers, please see below) (OR 0.64; 95% CI 0.46–0.89; *p* = 0.008) with lower PD risk. Multivariable MR analyses showed that the causal association between drinks per week and PD is unlikely due to confounding by smoking behavior. Finally, frailty analyses suggested that the causal effects of both alcohol intake and smoking continuation on PD risk estimated from MR analysis were not explained by the presence of survival bias alone. Increased alcohol intake had a protective effect over PD risk, with the alcohol dehydrogenase 1B (ADH1B) locus as a potential candidate for further investigation of the mechanisms underlying this association.

In summary, although the question of whether alcohol intake is protective or harmful for the occurrence of PD and its symptoms is still open in a strict scientific sense, the available data suggest no major effects in either direction.

## Nicotine

Cigarette smoking is associated with an inverse risk of developing Parkinson’s disease (PD), which was consistently proven in several studies (Hernán et al. 2002; Kiyohara and Kusuhara 2011; Ritz et al. 2007). Compared to never smokers, former smokers had a 20% reduced PD risk. For current smokers, the PD risk is reduced by half as shown in a recent epidemiologic study by Gallo et al. (2019).

Chen et al. showed, in a large epidemiological study, that long-term smoking is more important for this inverse association than smoking intensity (Chen et al. 2010). The underlying mechanism for this association is not yet understood. It is difficult to prove whether there is a biologically protective effect of cigarette smoking for this neurodegenerative disease, or whether there is a predisposition to a somehow altered nicotinergic–dopaminergic reward system in people who will develop PD, which might reduce the likelihood of them taking up smoking (De Biasi and Dani 2011). A 65-year follow-up trial of 30,000 male British practitioners revealed a 40% lower risk of PD compared with never smokers (Mappin-Kasirer et al. 2020). In this trial those who quit smoking between 0 and 9 years ago had a 29% lower risk compared to never smokers, those who quit smoking 10 or more years ago still had a 14% lower risk of PD. The effect of former smoking confers a reduced risk of PD in subjects who quit smoking up to 30 years before disease onset, as shown in the afore mentioned trial by Gallo et al. (2019). These findings suggest that there must be a relation years before disease onset, whether this might be a protective effect of tobacco smoke or a predisposition not to start smoking.

Several efforts to prove a putative effect of nicotine in PD have been made but could not show beneficial effects (Clemens et al. 1995; Ebersbach et al. 1999; Lemay et al. 2004; Vieregge et al. 2001). As there is no sporadic or inherited PD in non-human primates or other animals and we do not yet understand the mechanism of neurodegeneration in PD, a biological protective effect of nicotine cannot be specifically investigated in animal models. Even if there might be an effect of nicotine in 1-methyl-4-phenyl-1,2,3,6-tetrahydropyridine (MTPT) lesioned monkeys or 6-hydroxydopamin (6-OHDA) lesioned mice on levodopa-induced dyskinesias, it could not improve movement quality (Quik et al. 2009). Tanner et al. showed, that among genetically identical twins, smoking was also associated with a lower PD risk, which makes genetic factors unlikely to be a major confounder (Tanner et al. 2002). Lee et al. found in two population-based case–control studies with 513 patients and 1147 controls, two single nucleotide polymorphisms (SNPs), out of a total of nine, which interacted with smoking and PD. For carriers of minor alleles of these two SNPs, the inverse association of smoking with PD was less pronounced. The authors postulate that larger studies of SNPs which are involved in individual susceptibility to xenobiotics for the risk of PD, may help to identify biological pathways involved in the inverse association of smoking and PD (Lee et al. 2018). Interestingly, there is also an inverse association for passive smoking and PD risk, as found by Searles Nielsen et al. in a case–control study with 154 PD patients and 173 controls (Searles Nielsen et al. 2012). This would suggest a protective effect of tobacco smoke, rather than a biological predisposition which reduces the likelihood of taking up smoking (in subjects who will develop PD). The authors noted that these results might just reflect risk aversion or unpleasant response to tobacco smoke.

Ritz et al. asked former smokers in a case control study with 1,808 PD patients and 1,876 controls, how easy it was for them to stop smoking. Former smokers reporting that “quitting smoking was extremely difficult”, had a 31% reduced risk of developing PD, compared with those reporting, “Quitting smoking was easy”. They also found a reduced PD risk for those who ever used nicotine substitutes. They concluded form these observations that smoking cessation is part of pre-manifest PD like constipation, olfactory dysfunction or REM sleep behavior disorder and that nicotine reward is less strong in those who later develop PD rather than a neuroprotective effect of smoking (Ritz et al. 2014).

A population-based Korean cohort study found sex differences for current female smokers who had a 23% lower risk while current male smokers showed a 50% lower risk to develop PD compared to non-smokers (Kim et al. 2020). A protective effect of smoking assumed men would benefit more than woman would. On the other hand, if smoking is considered more socially acceptable in men, those women who might have a predisposition for PD with an altered nicotinergic–dopaminergic reward system, may be less likely to start smoking compared to men with the same predisposition.

The link between smoking and PD still remains unclear. Further efforts to clarify this question are required. To better understand neurodegeneration in PD, It would be beneficial if a neuroprotective chemical compound in cigarette smoke could be identified, or a biological mechanism with altered nicotinergic/dopaminergic response was revealed in subjects who will develop PD.

New Zealand has announced it will outlaw smoking for the next generation. If this smoking prohibition is successful, there might be a chance to clarify if smoking is neuroprotective, because if that is the case, the incidence of PD should rise, compared to other populations. Unfortunately, it will take years until a non-smoking generation reaches the typical PD onset age.

## Conclusion

Several reports indicate benefits from coffee drinking, smoking and physical exercise, while no clear conclusion can be drawn for alcohol intake. Chocolate consumption is increased in PD patients and seems to add to dopamine replacement therapy, while milk seems to increase PD risk. Sleep may play a yet unappreciated role but mechanisms remain to be elucidated. Moderate to vigorous physical activity is a protective intervention in males.
